# 1-Benzoyl-3-(pyridin-2-yl)-1*H*-pyrazole

**DOI:** 10.1107/S1600536811033368

**Published:** 2011-08-27

**Authors:** Alexander H. Shelton, Andrew Stephenson, Michael D. Ward, Mohammad B. Kassim

**Affiliations:** a Department of Chemistry, University of Sheffield, Sheffield S3 7HF, England; b School of Chemical Sciences & Food Technology, Faculty of Science & Technology, Universiti Kebangsaan Malaysia, 43600 Selangor, Malaysia; c Fuel Cell Institute, Universiti Kebangsaan Malaysia, 43600 Selangor, Malaysia

## Abstract

In the title compound, C_15_H_11_N_3_O, the dihedral angle betwen the heterocyclic rings is 9.23 (5)° and the dihedral angle between the benzoyl and pyrazole rings is 58.64 (5)°. In the crystal, inversion dimers linked by pairs of C—H⋯O hydrogen bonds generate *R*_2_^2^(10) loops. The dimers stack into a column running parallel to the *b*-axis direction.

## Related literature

For related structures and background, see: Jones *et al.* (1997 ▶); Adams *et al.* (2006 ▶); Al-abbasi & Kassim (2011 ▶). For reference bond lengths, see: Allen *et al.* (1987 ▶).
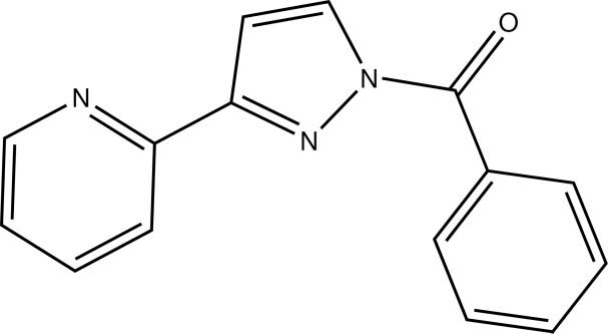


## Experimental

### 

#### Crystal data

C_15_H_11_N_3_O
*M* *_r_* = 249.27
Monoclinic, 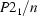

*a* = 10.6325 (11) Å
*b* = 5.7775 (6) Å
*c* = 19.572 (2) Å
β = 98.426 (6)°
*V* = 1189.3 (2) Å^3^
*Z* = 4
Mo *K*α radiation
μ = 0.09 mm^−1^
*T* = 296 K
0.20 × 0.15 × 0.10 mm

#### Data collection

Bruker SMART APEX CCD diffractometer
Absorption correction: multi-scan (*SADABS*; Bruker, 2000 ▶) *T*_min_ = 0.982, *T*_max_ = 0.991
10450 measured reflections
2735 independent reflections
2532 reflections with *I* > 2σ(*I*)
*R*_int_ = 0.023

#### Refinement

*R*[*F* ^2^ > 2σ(*F* ^2^)] = 0.035
*wR*(*F* ^2^) = 0.097
*S* = 1.00
2735 reflections
173 parameters
H-atom parameters constrained
Δρ_max_ = 0.35 e Å^−3^
Δρ_min_ = −0.22 e Å^−3^

### 

Data collection: *SMART* (Bruker, 2000 ▶); cell refinement: *SAINT* (Bruker, 2000 ▶); data reduction: *SAINT*; program(s) used to solve structure: *SHELXS97* (Sheldrick, 2008 ▶); program(s) used to refine structure: *SHELXL97* (Sheldrick, 2008 ▶); molecular graphics: *SHELXTL* (Sheldrick, 2008 ▶); software used to prepare material for publication: *SHELXTL*, *PARST* (Nardelli, 1995 ▶) and *PLATON* (Spek, 2009 ▶).

## Supplementary Material

Crystal structure: contains datablock(s) I, global. DOI: 10.1107/S1600536811033368/hb6370sup1.cif

Structure factors: contains datablock(s) I. DOI: 10.1107/S1600536811033368/hb6370Isup2.hkl

Supplementary material file. DOI: 10.1107/S1600536811033368/hb6370Isup3.cml

Additional supplementary materials:  crystallographic information; 3D view; checkCIF report

## Figures and Tables

**Table 1:** Hydrogen-bond geometry (Å, °)

*D*—H⋯*A*	*D*—H	H⋯*A*	*D*⋯*A*	*D*—H⋯*A*
C8—H8⋯O1^i^	0.93	2.44	3.3720 (13)	175

Symmetry code: (i) 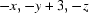.

**Fig. 1.**
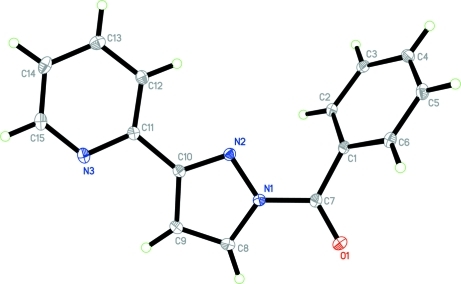
The molecular structure of the title compound with displacement ellipsoids drawn at the 30% probability level.

**Fig. 2.**
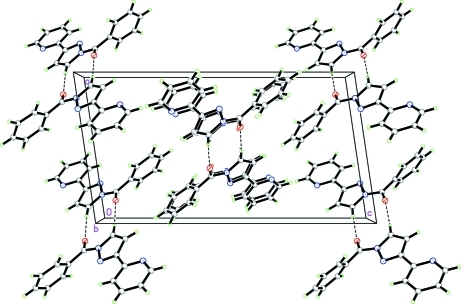
A packing diagram of the title compound viewing down the b-axis showing the intermolecular hydrogen bonds C—H···O (-x, 3 - y, -z).

## supplementary crystallographic information

### Comment

The starting material, 3-(2-pyridyl)pyrazole, is a bidentate ligand which is commonly used in coordination chemistry (Jones *et al.* 1997 & Adams *et al.* 2006). The title compound is made up of a 3-(2-pyridyl)pyrazole and benzoyl fragments. This new compound has a potential to be applied as a tridentate ligand (*ONN*) involving the O atom on the carbonyl group and the N atom on the pyrazole and pyridine rings.

In the crystal structure, the mean planes of acetamide (O1/N1/C1/C7) and the benzene (C1/C2/C3/C4/C5/C6) fragments make a dihedral angle of 49.54 (5)° with each other. The mean planes of the pyrazole and pyridyl rings are slightly twisted and make a dihedral 9.23 (5)°. The C7—O1 bond length 1.2117 (12) is slightly longer that of the C=O found in another benzoyl derivative, 1-ethyl-1-methyl-3-(2-nitrobenzoyl)thiourea (Al-abbasi & Kassim, 2011). Other bond lengths and angles within the compounds are in the normal ranges (Allen *et al.* 1987).

A C—H···O intermolecular hydrogen bond links adjacent molecules into centrosymmetric dimers forming a one dimensional column parallel to the *b*-axis.

### Experimental

3-(2-pyridyl)pyrazole (0.728 g, 5.0 mmol) was deprotonated by reacting with NaH (60% in mineral oil) in 30 ml of dry THF under N_2_ at room temperature for 2 h. Then, benzoyl chloride (0.702 g, 5.0 mmol) was added slowly to the mixture and the temperature was brought to reflux and left stirring for 4 hrs. The solvent was removed and the residue was re-dissolved in a minimum volume of DCM, washed 3 times with 30 ml of distilled water. The organic fraction was collected and dried with MgSO_4_, filtered and the solvent was removed *in vacuo*. Slow evaporation of acetone/DCM solution of the residue afforded colourless blocks of (I). Yield 78%.

### Refinement

All H atoms were positioned geometrically with C—H bond lengths in the range of 0.93 - 0.97 Å and refined in the riding model approximation with *U*_iso_(H)=1.2*U*_eq_(C).

### Crystal data

**Table:**

C_15_H_11_N_3_O	*F*(000) = 520
*M**_r_* = 249.27	*D*_x_ = 1.392 Mg m^−^^3^
Monoclinic, *P*2_1_/*n*	Mo *K*α radiation, λ = 0.71073 Å
Hall symbol: -P 2yn	Cell parameters from 7032 reflections
*a* = 10.6325 (11) Å	θ = 4.7–55.0°
*b* = 5.7775 (6) Å	µ = 0.09 mm^−^^1^
*c* = 19.572 (2) Å	*T* = 296 K
β = 98.426 (6)°	Block, colourless
*V* = 1189.3 (2) Å^3^	0.20 × 0.15 × 0.10 mm
*Z* = 4	

### Data collection

**Table:**

Bruker SMART APEX CCD diffractometer	2735 independent reflections
Radiation source: fine-focus sealed tube	2532 reflections with *I* > 2σ(*I*)
graphite	*R*_int_ = 0.023
ω scans	θ_max_ = 27.5°, θ_min_ = 2.1°
Absorption correction: multi-scan (*SADABS*; Bruker, 2000)	*h* = −13→13
*T*_min_ = 0.982, *T*_max_ = 0.991	*k* = −7→7
10450 measured reflections	*l* = −25→25

### Refinement

**Table:**

Refinement on *F*^2^	Secondary atom site location: difference Fourier map
Least-squares matrix: full	Hydrogen site location: inferred from neighbouring sites
*R*[*F*^2^ > 2σ(*F*^2^)] = 0.035	H-atom parameters constrained
*wR*(*F*^2^) = 0.097	*w* = 1/[σ^2^(*F*_o_^2^) + (0.0577*P*)^2^ + 0.430*P*] where *P* = (*F*_o_^2^ + 2*F*_c_^2^)/3
*S* = 1.00	(Δ/σ)_max_ < 0.001
2735 reflections	Δρ_max_ = 0.35 e Å^−^^3^
173 parameters	Δρ_min_ = −0.22 e Å^−^^3^
0 restraints	Extinction correction: *SHELXL97* (Sheldrick, 2008), Fc^*^=kFc[1+0.001xFc^2^λ^3^/sin(2θ)]^-1/4^
Primary atom site location: structure-invariant direct methods	Extinction coefficient: 0.025 (3)

### Special details

**Table:**

Geometry. All e.s.d.'s (except the e.s.d. in the dihedral angle between two l.s. planes) are estimated using the full covariance matrix. The cell e.s.d.'s are taken into account individually in the estimation of e.s.d.'s in distances, angles and torsion angles; correlations between e.s.d.'s in cell parameters are only used when they are defined by crystal symmetry. An approximate (isotropic) treatment of cell e.s.d.'s is used for estimating e.s.d.'s involving l.s. planes.
Refinement. Refinement of *F*^2^ against ALL reflections. The weighted *R*-factor *wR* and goodness of fit *S* are based on *F*^2^, conventional *R*-factors *R* are based on *F*, with *F* set to zero for negative *F*^2^. The threshold expression of *F*^2^ > σ(*F*^2^) is used only for calculating *R*-factors(gt) *etc*. and is not relevant to the choice of reflections for refinement. *R*-factors based on *F*^2^ are statistically about twice as large as those based on *F*, and *R*- factors based on ALL data will be even larger.

### Fractional atomic coordinates and isotropic or equivalent isotropic displacement parameters (Å^2^)

**Table:**

	*x*	*y*	*z*	*U*_iso_*/*U*_eq_	
O1	0.13720 (7)	1.43117 (13)	0.06519 (4)	0.02310 (19)	
N1	0.16990 (8)	1.10288 (14)	0.00732 (4)	0.01636 (19)	
N2	0.24411 (8)	0.91307 (15)	−0.00018 (4)	0.01622 (19)	
N3	0.22475 (8)	0.59436 (16)	−0.15957 (4)	0.0200 (2)	
C4	0.43069 (10)	1.04513 (19)	0.25014 (5)	0.0206 (2)	
H4	0.4799	1.0072	0.2919	0.025*	
C3	0.34100 (10)	0.88896 (18)	0.21845 (5)	0.0187 (2)	
H3	0.3304	0.7468	0.2392	0.022*	
C2	0.26702 (9)	0.94408 (18)	0.15592 (5)	0.0172 (2)	
H2	0.2096	0.8371	0.1338	0.021*	
C1	0.27989 (9)	1.16160 (17)	0.12676 (5)	0.0162 (2)	
C7	0.19083 (9)	1.24595 (17)	0.06590 (5)	0.0169 (2)	
C10	0.20214 (9)	0.83729 (17)	−0.06333 (5)	0.0158 (2)	
C11	0.26054 (9)	0.63418 (17)	−0.09172 (5)	0.0163 (2)	
C15	0.27577 (11)	0.4098 (2)	−0.18654 (5)	0.0231 (2)	
H15	0.2519	0.3799	−0.2333	0.028*	
C14	0.36167 (10)	0.26093 (19)	−0.14932 (6)	0.0231 (2)	
H14	0.3938	0.1344	−0.1705	0.028*	
C5	0.44676 (10)	1.25791 (19)	0.21942 (5)	0.0209 (2)	
H5	0.5091	1.3597	0.2397	0.025*	
C6	0.36981 (10)	1.31859 (18)	0.15852 (5)	0.0189 (2)	
H6	0.3781	1.4634	0.1389	0.023*	
C13	0.39894 (10)	0.30481 (19)	−0.07940 (6)	0.0216 (2)	
H13	0.4570	0.2089	−0.0529	0.026*	
C12	0.34759 (9)	0.49456 (18)	−0.05019 (5)	0.0186 (2)	
H12	0.3708	0.5284	−0.0036	0.022*	
C9	0.10122 (9)	0.97734 (18)	−0.09703 (5)	0.0187 (2)	
H9	0.0569	0.9577	−0.1413	0.022*	
C8	0.08350 (9)	1.14563 (18)	−0.05088 (5)	0.0185 (2)	
H8	0.0247	1.2657	−0.0573	0.022*	

### Atomic displacement parameters (Å^2^)

**Table:**

	*U*^11^	*U*^22^	*U*^33^	*U*^12^	*U*^13^	*U*^23^
O1	0.0271 (4)	0.0165 (4)	0.0252 (4)	0.0037 (3)	0.0025 (3)	−0.0009 (3)
N1	0.0175 (4)	0.0150 (4)	0.0165 (4)	0.0007 (3)	0.0020 (3)	0.0007 (3)
N2	0.0181 (4)	0.0141 (4)	0.0167 (4)	0.0002 (3)	0.0031 (3)	−0.0001 (3)
N3	0.0232 (4)	0.0211 (5)	0.0159 (4)	−0.0032 (3)	0.0029 (3)	−0.0016 (3)
C4	0.0183 (5)	0.0258 (5)	0.0175 (5)	0.0038 (4)	0.0021 (4)	−0.0022 (4)
C3	0.0211 (5)	0.0173 (5)	0.0184 (5)	0.0032 (4)	0.0055 (4)	0.0011 (4)
C2	0.0183 (5)	0.0155 (5)	0.0181 (5)	−0.0010 (4)	0.0039 (4)	−0.0024 (4)
C1	0.0181 (4)	0.0162 (5)	0.0150 (4)	0.0003 (4)	0.0047 (3)	−0.0022 (4)
C7	0.0187 (5)	0.0151 (5)	0.0177 (4)	−0.0018 (4)	0.0050 (4)	−0.0003 (4)
C10	0.0167 (4)	0.0161 (5)	0.0148 (4)	−0.0027 (4)	0.0025 (3)	0.0014 (4)
C11	0.0162 (4)	0.0168 (5)	0.0162 (4)	−0.0035 (4)	0.0035 (3)	−0.0005 (4)
C15	0.0265 (5)	0.0248 (5)	0.0189 (5)	−0.0055 (4)	0.0066 (4)	−0.0049 (4)
C14	0.0217 (5)	0.0205 (5)	0.0290 (5)	−0.0035 (4)	0.0105 (4)	−0.0065 (4)
C5	0.0181 (5)	0.0233 (5)	0.0216 (5)	−0.0034 (4)	0.0038 (4)	−0.0062 (4)
C6	0.0218 (5)	0.0161 (5)	0.0198 (5)	−0.0024 (4)	0.0066 (4)	−0.0022 (4)
C13	0.0170 (5)	0.0200 (5)	0.0281 (5)	−0.0009 (4)	0.0039 (4)	0.0006 (4)
C12	0.0179 (4)	0.0198 (5)	0.0178 (5)	−0.0027 (4)	0.0020 (3)	−0.0006 (4)
C9	0.0179 (5)	0.0209 (5)	0.0168 (4)	−0.0008 (4)	0.0010 (4)	0.0019 (4)
C8	0.0169 (4)	0.0190 (5)	0.0192 (5)	0.0000 (4)	0.0010 (4)	0.0033 (4)

### Geometric parameters (Å, °)

**Table:**

O1—C7	1.2116 (12)	C10—C9	1.4260 (14)
N1—N2	1.3713 (12)	C10—C11	1.4740 (14)
N1—C8	1.3768 (12)	C11—C12	1.3955 (14)
N1—C7	1.4045 (13)	C15—C14	1.3819 (16)
N2—C10	1.3255 (12)	C15—H15	0.9300
N3—C15	1.3393 (14)	C14—C13	1.3910 (15)
N3—C11	1.3465 (12)	C14—H14	0.9300
C4—C5	1.3900 (15)	C5—C6	1.3883 (14)
C4—C3	1.3910 (15)	C5—H5	0.9300
C4—H4	0.9300	C6—H6	0.9300
C3—C2	1.3913 (14)	C13—C12	1.3850 (15)
C3—H3	0.9300	C13—H13	0.9300
C2—C1	1.3952 (14)	C12—H12	0.9300
C2—H2	0.9300	C9—C8	1.3589 (15)
C1—C6	1.3961 (14)	C9—H9	0.9300
C1—C7	1.4908 (13)	C8—H8	0.9300
			
N2—N1—C8	112.31 (8)	C12—C11—C10	121.39 (9)
N2—N1—C7	122.22 (8)	N3—C15—C14	124.21 (10)
C8—N1—C7	125.20 (9)	N3—C15—H15	117.9
C10—N2—N1	104.10 (8)	C14—C15—H15	117.9
C15—N3—C11	116.90 (9)	C15—C14—C13	118.44 (10)
C5—C4—C3	120.04 (9)	C15—C14—H14	120.8
C5—C4—H4	120.0	C13—C14—H14	120.8
C3—C4—H4	120.0	C6—C5—C4	120.02 (10)
C4—C3—C2	120.40 (10)	C6—C5—H5	120.0
C4—C3—H3	119.8	C4—C5—H5	120.0
C2—C3—H3	119.8	C5—C6—C1	119.82 (10)
C3—C2—C1	119.29 (9)	C5—C6—H6	120.1
C3—C2—H2	120.4	C1—C6—H6	120.1
C1—C2—H2	120.4	C12—C13—C14	118.53 (10)
C2—C1—C6	120.32 (9)	C12—C13—H13	120.7
C2—C1—C7	122.17 (9)	C14—C13—H13	120.7
C6—C1—C7	117.18 (9)	C13—C12—C11	119.02 (9)
O1—C7—N1	119.50 (9)	C13—C12—H12	120.5
O1—C7—C1	122.72 (9)	C11—C12—H12	120.5
N1—C7—C1	117.77 (9)	C8—C9—C10	105.47 (9)
N2—C10—C9	111.81 (9)	C8—C9—H9	127.3
N2—C10—C11	120.77 (9)	C10—C9—H9	127.3
C9—C10—C11	127.41 (9)	C9—C8—N1	106.31 (9)
N3—C11—C12	122.90 (9)	C9—C8—H8	126.8
N3—C11—C10	115.72 (9)	N1—C8—H8	126.8

### Hydrogen-bond geometry (Å, °)

**Table:**

*D*—H···*A*	*D*—H	H···*A*	*D*···*A*	*D*—H···*A*
C8—H8···O1^i^	0.93	2.44	3.3720 (13)	175

Symmetry codes: (i) −*x*, −*y*+3, −*z*.
